# The Effects of Anxiety and Self-Control on Smartphone Addiction Among Children and Adolescents at Risk for Depression

**DOI:** 10.3390/healthcare14080990

**Published:** 2026-04-09

**Authors:** Miseon Kwak, Eunju Bae, Wonjae Choi, Myung Ho Lim

**Affiliations:** 1 Department of Psychology, Graduate School, Dankook University, Cheonan 31116, Republic of Korea; msk8958@dankook.ac.kr (M.K.); zzazzacnamu@naver.com (E.B.); 2 Department of Visual Communication Design, College of Music and Arts, Dankook University, Yongin 16890, Republic of Korea; 3 Department of Psychology and Psychotherapy, College of Health Science, Dankook University, Cheonan 31116, Republic of Korea

**Keywords:** smartphone addiction, anxiety, aggression, school bullying, self-esteem, I-PACE model

## Abstract

**Background/Objectives**: Research comprehensively analyzing the psychological characteristics and factors related to smartphone addiction in Korean children and adolescents at risk for depression remains scarce. This study utilized large-scale cohort data to examine the differences in psychological characteristics between an at-risk group for depression and a control group, and to identify the specific factors influencing smartphone addiction within the at-risk group. **Methods**: Data were obtained from the school-based cohort of internet and smartphone users conducted by the National Center for Mental Health (NCMH), involving a total of 2294 children and adolescents (1009 in the at-risk for depression group and 1285 in the control group). Assessment tools included the Children’s Depression Inventory (CDI), State-Trait Anxiety Inventory for Children (STAIC/TAIC), Self-Esteem Scale (SES), Self-Control Scale, Aggression Questionnaire (K-AQ), School Bullying (SB) scale, and the Smartphone Addiction Scale-Short Form (SAS-SV). **Results**: Analysis of Covariance (ANCOVA) revealed that the at-risk group exhibited significantly higher levels of anxiety, aggression, involvement in school bullying, and smartphone addiction compared to the control group, while showing lower levels of self-esteem and self-control. Furthermore, multiple regression analysis indicated that higher anxiety and lower self-control were significant predictors of increased smartphone addiction levels. **Conclusions**: These findings support the Interaction of Person-Affect-Cognition-Execution (I-PACE) model, which posits that emotional vulnerability and deficits in executive functions lead to addictive behaviors. The results suggest that reducing anxiety and enhancing self-control are critical factors in the prevention of smartphone addiction among children and adolescents.

## 1. Introduction

Adolescence is a critical developmental period characterized by rapid physical, emotional, and social changes [1]. According to the World Health Organization, the mental health of adolescents is fundamental to their overall well-being and significantly influences outcomes in adulthood [2]. Children and adolescents with mental health issues often continue to experience mental health disorders, lower life satisfaction, and poor health-related quality of life as they transition into adulthood [3]. While the primary developmental task in late childhood (typically the upper elementary years) is to cultivate a sense of competence through academic achievements and peer relationships, the focus shifts in adolescence (middle and high school years) to the establishment of self-identity and the exploration of career and value systems [4]. Academic issues are a universal concern for adolescents worldwide; however, the excessive academic pressure to achieve high scholastic performance is particularly pronounced in South Korea [5].

Globally, a significant portion of adolescents experiences mental disorders, with depression and anxiety being the most prevalent [6]. Over the past decade, the prevalence of adolescent depression has been increasing worldwide [7]. Depressive symptoms persisting from childhood through adolescence further exacerbate adult outcomes [8], specifically, the chronic depression group shows a higher tendency toward anxiety disorders, multiple mental health issues, suicide attempts, and treatment-seeking behavior compared to the transient depression group. Furthermore, they are more likely to progress toward chronic and recurrent depression in adulthood [9]. Risk factors for depression include family history, exposure to bullying, and negative home environments [10]. Psychosocial risk factors, such as those related to family and school environments, can contribute significantly to the increasing rate of depressive symptoms in adolescents [11].

According to a 2015 national survey, smartphone ownership rates among Korean children and adolescents had already reached a high level across all school grades, with a majority of adolescents reported to be using smartphones on a daily basis [12]. This trend has persisted until recently. The smartphone usage rate among South Korean adolescents on weekdays significantly increased from 96.4% in 2020 to 97.1% in 2023. During both 2020 and 2023, the average smartphone usage time remained consistent at 4.7 h on weekdays and 6.6 h on weekends. By 2023, the proportion of adolescents exhibiting high levels of overdependence reached 28% [13]. Lin et al. [14] proposed compulsive use, tolerance, withdrawal, and functional impairment as core components of smartphone addiction. They suggested that problematic use should be viewed as clinically significant when users find it difficult to self-regulate the duration and frequency of use (compulsive use), require increasingly frequent or prolonged use to experience satisfaction (tolerance), experience negative emotions such as anxiety, restlessness, and depression when use is restricted or discontinued (withdrawal), and suffer substantial impairment in daily functional areas such as academics, interpersonal relationships, sleep, and occupational performance (functional impairment). Meanwhile, in the DSM-5 and ICD-11, the behavioral addictions currently recognized officially are very limited, such as gambling disorder, and smartphone addiction is not yet included as an independent diagnostic category [15]. Nevertheless, various studies have suggested that smartphone use can be conceptualized as a form of behavioral addiction, given that it involves patterns of repetitive and loss-of-control use, as well as clinically significant functional impairment [16]. Problematic smartphone use has been found to be more prevalent among younger individuals, females, and those with higher educational backgrounds [17].

Furthermore, the excessive use of smartphones is closely associated with various psychological and behavioral disorders [18]. While motivations for gaming and social networking can serve as risk factors for smartphone addiction, healthy peer relationships may act as a protective factor [19]. Research has also indicated that authoritarian parenting styles, low self-control, and low self-esteem increase the risk of problematic use, whereas academic motivation and achievement can mitigate these risks. Scores for depression and anxiety have been identified as independent positive predictors of smartphone addiction. Additionally, it has been reported that young adults with Type A personalities, who experience high stress levels and low mood, may be highly vulnerable to smartphone addiction due to a lack of positive stress-coping mechanisms and mood-management skills [20].

Anxiety disorders and depression are frequently comorbid in children and adolescents, a phenomenon largely attributed to the shared domain of negative affectivity. Specifically, 25% to 50% of adolescents with depression present with comorbid anxiety disorders, while approximately 10% to 15% of adolescents with anxiety experience comorbid depression [21]. High levels of smartphone addiction have been significantly associated with lower cognitive function and increased physical aggression, verbal aggression, anger, and hostility [22]. Furthermore, research has indicated that both cyberbullying victimization and perpetration exert a significant mediating effect on the relationship between mobile phone addiction and depression among high school students [23]. Additionally, experiences of violent treatment have been reported as significant predictors of smartphone overdependence [24].

The Interaction of Person-Affect-Cognition-Execution (I-PACE) model [25] is applicable to various types of addictive behaviors, including gambling, gaming, buying-shopping, and compulsive sexual behavior disorders. This model posits that addictive behaviors emerge from the interaction between predisposing factors, affective and cognitive responses to specific stimuli, and executive functions such as inhibitory control and decision-making. Within the process of addictive behavior, the association between cue-reactivity/craving and diminished inhibitory control contributes to the development of habitual behaviors.

Despite these theoretical foundations, research simultaneously considering anxiety, self-esteem (affective factors), self-control, aggression (executive and behavioral regulation factors), and bullying experiences (environmental and social context factors) specifically in adolescents at risk for depression remains limited. In particular, there is a lack of studies involving South Korean children and adolescents that compare depression-risk groups with control groups to examine differences in psychosocial factors related to smartphone overdependence. Therefore, this study aims to identify the differences in psychological characteristics between children and adolescents at risk for depression and a control group, and furthermore, to explore the specific factors influencing smartphone addiction within the depression-risk group.

## 2. Materials and Methods

### 2.1. Participants and Ethics Statement

This study utilized data from the “School-Based Internet, Gaming, and Smartphone User Cohort (iCURE),” established as part of the Mental Health R&D project by the National Center for Mental Health (NCMH). The original cohort study was conducted in accordance with the Declaration of Helsinki, and written informed consent was obtained from all participants and their parents or legal guardians prior to enrollment. The primary data collection was approved by the Institutional Review Board (IRB) of the Catholic University of Korea (MC140NM10085). The findings of this study are those of the authors and do not necessarily reflect the official views of the NCMH. Based on the median score of children depression inventory (CDI) (7 points) across the entire sample, participants with scores equal to or higher than the median were classified into the “depression-risk group,” while those with lower scores were assigned to the “control group.” We referred to the methodology of previous studies that categorized depression symptom scales or related indicators using a median split for analysis [26]. The final study population consisted of 2294 participants, including 1009 in the depression-risk group and 1285 in the control group.

The data were collected between 2015 and 2016. Age was determined based on the year of birth, and international age (Man-age) was calculated relative to 2015, the initial year of the study. It should be noted that a discrepancy of approximately one year in actual age may occur depending on the specific timing of the survey. This study was reviewed and approved by the Institutional Review Board (IRB) of Dankook University (DKU IRB 2025-06-030). which waived the requirement for informed consent because it involved the secondary analysis of de-identified, publicly available data.

### 2.2. Measures

#### 2.2.1. Children’s Depression Inventory (CDI)

To assess depressive symptoms in children, this study utilized the Children’s Depression Inventory (CDI). Originally developed by Kovacs [27] by adapting Beck’s Depression Inventory (BDI) [28] for children, the CDI was later translated and validated for the Korean population by Cho and Lee [29]. The scale is designed for children and adolescents aged 7 to 17 and consists of 27 items. Participants evaluate their emotional state over the past two weeks using a self-report format on a 3-point Likert scale (0–2 points). Total scores range from 0 to 54, with higher scores indicating greater severity of depression. In the validation study of the Korean version of the CDI, the instrument demonstrated favorable reliability, with a test–retest reliability of 0.82, a split-half reliability of 0.71, and an internal consistency coefficient (Cronbach’s alpha) of 0.88.

#### 2.2.2. Trait Anxiety Inventory for Children (TAIC)

To assess trait anxiety in children, this study utilized the Korean version of the Trait Anxiety Inventory for Children (TAIC). The TAIC is a standardized adaptation by Cho and Choi [30] of the State-Trait Anxiety Inventory for Children (STAIC) originally developed by Spielberger [31]. The scale consists of 20 items rated on a 3-point Likert scale, with total scores ranging from 20 to 60. Regarding the interpretation of scores, a range of 39–42 indicates a slightly high level of trait anxiety, 43–46 suggests a considerably high level, and 47 or higher signifies a very high level. In the study by Cho and Choi, the instrument demonstrated a high level of internal consistency, with a Cronbach’s alpha of 0.85.

#### 2.2.3. Self-Esteem Scale (SES)

The Self-Esteem Scale (SES), originally developed by Rosenberg [32], measures an individual’s degree of self-respect and self-acceptance to assess global self-esteem. Following its initial development in 1965, Rosenberg revised and updated the scale in 1989, making it publicly available without copyright restrictions. The scale has been translated and adapted into Korean by numerous researchers, including Lee and Lee [33], Lee [34], and Jon [35]. This self-report instrument consists of 10 items: five items measuring positive self-esteem and five items measuring negative self-esteem. Each item is rated on a 4-point Likert scale (0–3 points), where higher total scores reflect a higher level of self-esteem.

#### 2.2.4. Self-Control Scale

To measure self-control, this study utilized a scale reconstructed by Nam and Ok [36], based on the original instrument developed by Gottfredson and Hirschi [37]. This scale consists of 20 items designed to assess an individual’s ability to engage in deliberate thought before acting, delay immediate gratification for more effective problem-solving, and identify tendencies such as impulsivity, self-centeredness, and a preference for physical action over verbal communication. Participants responded on a 5-point Likert scale (1 = “Not at all,” 2 = “Sometimes,” 3 = “Neutral,” 4 = “Often,” and 5 = “Always”). The mean score of all 20 items was used as the variable for self-control, with higher scores indicating a higher level of self-control. In the current sample, the reliability (Cronbach’s alpha) of the self-control scale was 0.746.

#### 2.2.5. Korean Version of the Aggression Questionnaire (K-AQ)

The level of aggression was assessed using the Korean version of the Aggression Questionnaire (K-AQ), translated and validated by Seo and Kwon [38] from the original Aggression Questionnaire developed by Buss and Perry [39]. The scale comprises 27 items categorized into four sub-factors: physical aggression, verbal aggression, anger, and hostility. Responses are recorded on a 5-point Likert scale ranging from 1 (Not at all) to 5 (Very much so), with total scores ranging from 27 to 135. Higher scores reflect a higher propensity for aggression. The internal consistency (Cronbach’s alpha) of the scale in this study was 0.88.

#### 2.2.6. School Bullying (SB) Scale

To evaluate the risk level of school bullying victimization, this study utilized the School Bullying Scale employed by the Seoul Child and Adolescent Mental Health Center [40]. The SB scale is a 12-item self-report instrument designed to assess the absence of close peer relationships and experiences of being bullied at school. It employs a 4-point Likert scale (e.g., “No,” “A little,” “Considerably,” and “Very much”). The total score is calculated by summing the number of items for which the respondent answered “Considerably” or “Very much.” According to the interpretation guidelines, a total count of two or fewer items indicates a likelihood of transient bullying, three to seven items suggest a risk of being bullied, and eight or more items indicate a very high risk, necessitating professional counseling [41].

#### 2.2.7. Smartphone Addiction Scale–Short Version (SAS-SV)

Smartphone addiction levels were measured using the Smartphone Addiction Scale–Short Version (SAS-SV), developed by Kwon et al. [42]. The SAS-SV is a 10-item abbreviated tool, with each item rated on a 6-point Likert scale ranging from 1 (Strongly disagree) to 6 (Strongly agree). This scale assesses core elements of smartphone overdependence, including absorption, loss of control, interference with daily life, withdrawal, and tolerance. Higher total scores indicate a greater risk of smartphone addiction. Based on Korean adolescent standards, individuals are classified into the high-risk group if they score 31 or higher for males and 33 or higher for females.

### 2.3. Statistical Analysis

The collected data were analyzed using R version 4.4.5. First, Chi-square testswere performed to examine the demographic characteristics of the participants, such as gender and age. Second, an Analysis of Covariance (ANCOVA), controlling for gender, was conducted to compare the scores of anxiety, self-esteem, self-control, bullying, aggression, and smartphone addiction between the depression-risk group and the control group. Furthermore, multiple regression analysis was performed within the depression-risk group to identify the influence of anxiety, self-esteem, self-control, bullying, and aggression on smartphone addiction. For all statistical tests, a *p*-value of less than 0.05 was considered statistically significant.

## 3. Results

### 3.1. General Characteristics of Participants

There was no statistically significant difference in age between the depression-risk group and the control group (M = 11.91, SD = 1.21 for the depression-risk group; M = 11.82, SD = 1.21 for the control group). However, a significant difference was observed in gender distribution. Within the depression-risk group (n = 1009), 506 were male (50.1%) and 503 were female (49.9%). In contrast, within the control group (n = 1285), 801 were male (62.3%) and 484 were female (37.7%). These results indicate a higher proportion of males in the control group and a higher proportion of females in the depression-risk group compared to the control group. Additionally, there was a significant difference in CDI scores between the two groups (t = −56.14, *p* < 0.01), with the depression-risk group reporting a higher mean score (M = 14.33, SD = 6.43) than the control group (M = 3.46, SD = 2.31) (Table 1).

### 3.2. Differences in Psychological Characteristics Between the Depression-Risk and Control Groups

The results of the ANCOVA, controlling for gender, revealed significant differences between the depression-risk and control groups across multiple scales. Compared to the control group, the depression-risk group scored significantly higher in trait anxiety, aggression, school bullying experiences, and smartphone addiction, while scoring significantly lower in self-esteem and self-control. Specifically, trait anxiety scores were significantly higher in the depression-risk group (35.07 ± 7.47) than in the control group (25.37 ± 4.82; F = 1431.33, *p* < 0.001). Aggression scores were also significantly higher in the depression-risk group (63.37 ± 16.39) compared to the control group (53.34 ± 12.98; F = 273.62, *p* < 0.001). Similarly, school bullying scores were significantly higher in the depression-risk group (14.26 ± 3.51) than in the control group (12.62 ± 1.34; F = 236.56, *p* < 0.001), and smartphone addiction scores were confirmed to be significantly higher in the depression-risk group (26.83 ± 10.66) than in the control group (18.61 ± 7.91; F = 455.24, *p* < 0.001). In contrast, self-esteem scores were significantly lower in the depression-risk group (27.78 ± 5.23) than in the control group (33.68 ± 4.19; F = 899.23, *p* < 0.001). Likewise, self-control scores were significantly lower in the depression-risk group (57.23 ± 6.91) compared to the control group (63.93 ± 7.91; F = 458.51, *p* < 0.001) (Table 2).

### 3.3. Factors Influencing Smartphone Addiction in the Depression-Risk Group

A multiple regression analysis was conducted to identify the factors influencing smartphone addiction specifically within the depression-risk group. The results indicated that trait anxiety scores had a statistically significant positive (+) effect on smartphone addiction (B = 0.40, beta = 0.28, t = 8.38, *p* < 0.001). Conversely, self-control scores were confirmed to have a statistically significant negative (-) effect on smartphone addiction (B = −0.42, beta = −0.27, t = −8.13, *p* < 0.001). On the other hand, self-esteem (B = 0.02, beta = 0.01, t = 0.22, *p* = 0.826), aggression (B = −0.00, beta = −0.00, t = −0.01, *p* = 0.989), and school bullying scores (B = −0.06, beta = −0.02, t = −0.57, *p* = 0.566) did not show a statistically significant influence on smartphone addiction. The explanatory power of the regression model for the depression-risk group was 17.7%, contributing to the explanation of a certain portion of the variance in smartphone addiction (Adj. R2 = 0.177, *p* < 0.001) (Table 3).

## 4. Conclusions and Discussion

Regarding gender distribution, the results of this study showed a higher proportion of females in the depression-risk group, whereas a higher proportion of males was observed in the control group. This finding is consistent with previous research reporting that adolescent females experience higher levels of internalizing problems, such as depression, compared to their male counterparts [21].

This study aimed to identify differences in psychological characteristics between the depression-risk and control groups of children and adolescents and to explore the specific factors influencing smartphone addiction within the depression-risk group.

The analysis revealed significant differences between the depression-risk and control groups across various measures, including trait anxiety, self-esteem, self-control, aggression, school bullying, and smartphone addiction. Specifically, the depression-risk group exhibited higher levels of anxiety, aggression, school bullying, and smartphone addiction compared to the control group, while showing lower levels of self-esteem and self-control. These findings suggest that children and adolescents at risk for depression are emotionally vulnerable, possess insufficient self-regulatory abilities, and are more likely to exhibit maladaptive patterns in their smartphone usage.

These findings are consistent with previous studies that have reported associations among depression, anxiety, low self-control during adolescence. First, the observation that the depression-risk group exhibited higher anxiety and lower self-esteem than the control group supports the concept of emotional vulnerability associated with depression. According to one study [43], female adolescents with comorbid depression and anxiety experienced more severe alienation from peers and family compared to those presenting with only depressive or anxious symptoms.

On the other hand, the low self-control observed in the depression-risk group suggest that internalizing problems (depression) and externalizing problems in adolescents coexist and interact with one another. A study [44] conducted in Spain reported a significant correlation between low self-control scores and the exacerbation of anxiety-depressive symptoms, highlighting the importance of fostering self-control to prevent internet addiction, substance use, and anxiety-depression among adolescents and young adults.

While significant differences were observed across all variables between the depression-risk and control groups, the multiple regression analysis specifically for the depression-risk group revealed that only anxiety and self-control exerted a significant association on smartphone addiction.

This is consistent with a longitudinal study [45] of 582 German adolescents, which reported social anxiety as a risk factor for smartphone overuse. It also aligns with a large-scale Korean study [24] of 53,457 adolescents, which found that those with high anxiety levels were more than three times more likely to be at risk for smartphone overdependence compared to their low-anxiety counterparts. Furthermore, our findings correspond with research [46] on Canadian adolescents’ problematic smartphone use trajectories, where higher self-regulation was associated with lower usage levels, and with European studies indicating that increased self-control reduces smartphone addiction [47].

However, our results diverge from previous research [48] and meta-analyses [49] suggesting that lower self-esteem increases the likelihood of smartphone addiction. Additionally, they contrast with studies on Turkish and Korean adolescents that reported a positive impact of aggressive behavior and high aggression on smartphone addiction levels [50,51]. Our findings also do not align with longitudinal research indicating that school bullying victimization significantly predicts mobile phone addiction or reports of a bidirectional link between peer harassment and phone addiction [52,53].

These discrepancies suggest that while aggression, self-esteem, and bullying are important characteristics of the depression-risk group, anxiety (as an emotional motivator) and self-control (as a behavioral regulation mechanism) may have absorbed the variance of other variables or emerged as the most potent determinants in predicting smartphone addiction within this specific population.

Children and adolescents experiencing depression may utilize smartphones to escape from real-life stress or seek psychological comfort in online spaces. However, when such usage becomes repetitive, it delays the resolution of real-world problems and increases the likelihood of a vicious cycle characterized by further diminished control over smartphone use. The significant association of high anxiety and low self-control on smartphone addiction identified in this study suggests that this maladaptive cycle may be particularly pronounced among the depression-risk group.

Furthermore, these findings are consistent with the theoretical framework of the I-PACE model. This model posits that digital addiction results from the interaction between an individual’s psychological vulnerabilities (e.g., anxiety, low self-esteem), deficits in executive functions (e.g., low self-control, aggression), and environmental/social context factors (e.g., bullying), which together trigger problematic internet use behaviors. In this context, anxiety acts as a driver to facilitate smartphone use to alleviate negative affect, while low self-control represents an inability to regulate such problematic usage, ultimately leading to addiction. The results of our multiple regression analysis—showing that higher anxiety and lower self-control predict higher levels of smartphone addiction—provide empirical support for these theoretical mechanisms within the depression-risk population.

Despite its contributions, this study has several limitations. First, all research variables were measured using self-report questionnaires, which may have led to the underestimation or overestimation of responses due to social desirability or subjective perception. Second, the depression-risk group was categorized arbitrarily based on the median score rather than clinical cut-off points. Since this criterion may not perfectly align with clinically diagnosed groups, future research should utilize diagnostic assessments or established clinical cut-off points to define depression risk. Nevertheless, this study holds exploratory significance in examining the characteristics of a relative risk group within a large-scale sample. Third, due to the limited measurement of individual-specific traits, the findings might have been association by participants’ personal characteristics, as well as various environmental and situational factors. Fourth, the regression model has a relatively low explanatory power, suggesting that other unmeasured factors may influence the results. Despite these limitations, this study has significant practical implications as it identifies the psychological characteristics of depression-risk children and adolescents and explores the factors influencing smartphone addiction within a large-scale Korean sample.

In conclusion, this study confirmed that children and adolescents in the depression-risk group exhibited higher levels of anxiety, aggression, school bullying, and smartphone addiction, alongside lower levels of self-esteem and self-control, compared to the control group. Notably, the findings highlighted that high anxiety and low self-control significantly association smartphone addiction within the depression-risk population. These results provide foundational data for developing educational programs and refining institutional policies aimed at mitigating the addictive use of digital media among youth experiencing emotional difficulties. Future research should strive to expand and validate these findings through diverse research designs and methodologies.

## Figures and Tables

**Table 1:** Epidemiological characteristics between Depression Risk Group and comparison group.

Variables	Depression-Risk Group (n = 1009)	Control Group (n = 1285)	t/χ^2^	*p* Value
Age	11.91 ± 1.21	11.82 ± 1.21	−1.61	0.11
Sex			9.72 *	*p* < 0.01
Male	506 (50.1)	801 (62.3)		
Female	503 (49.9)	484 (37.7)		
CDI (depression)	14.33 ± 6.43	3.46 ± 2.31	−56.14 *	*p* < 0.01

Data represent mean ± standard deviation, by independent *t*-test, or n (%), by chi-square test, * *p* < 0.05.

**Table 2:** Comparison of Psychosocial Variables Between the Depression-Risk and Control Groups (ANCOVA).

Sortation	Depression-Risk Group (n = 1009)	Control Group (n = 1285)	*F*	*p*
	M ± SD	M ± SD		
State-Trait Anxiety	35.07 ± 7.47	25.37 ± 4.82	1431.333 *	<0.001
Self-Esteem	27.78 ± 5.23	33.68 ± 4.19	899.230 *	<0.001
Self-Control	57.23 ± 6.91	63.93 ± 7.91	458.512 *	<0.001
Aggression	63.37 ± 16.39	53.34 ± 12.98	273.620 *	<0.001
School Bullying	14.26 ± 3.51	12.62 ± 1.34	236.564 *	<0.001
Smartphone Addiction	26.83 ± 10.66	18.61 ± 7.91	455.243 *	<0.001

* *p* < 0.05.

**Table 3:** Effects of Psychosocial Factors on Smartphone Addiction in the Depression-Risk Group.

Sortation	*B*	*S.E*	*β*	*t*	*p*
**State-Trait Anxiety**	0.399	0.048	0.280	8.381 *	<0.001
**Self-Esteem**	0.015	0.069	0.007	0.220	0.826
**Self-Control**	−0.415	0.051	−0.270	−8.130	<0.001
**Aggression**	<−0.001	0.021	<−0.001	−0.014	0.989
**School Bullying**	−0.056	0.097	0.018	−0.574	0.566
*R*^2^ = 0.181, Adjusted *R*^2^ = 0.177, *F* (*p*) = 43.310 (<0.001)					

* *p* < 0.05, *B* = Unstandardized coefficient; *S.E* = Standard error; β = Standardized coefficient.

## Data Availability

Restrictions apply to the availability of these data. Data were obtained from third party data and are available with the permission of third party.
